# Tuberculosis — United States, 2021

**DOI:** 10.15585/mmwr.mm7112a1

**Published:** 2022-03-25

**Authors:** Thomas D. Filardo, Pei-Jean Feng, Robert H. Pratt, Sandy F. Price, Julie L. Self

**Affiliations:** ^1^Epidemic Intelligence Service, CDC; ^2^Division of Tuberculosis Elimination, National Center for HIV, Viral Hepatitis, STD, and TB Prevention, CDC.

During 1993–2019, the incidence of tuberculosis (TB) in the United States decreased steadily; however, during the later years of that period the annual rate of decline slowed (*1*) until 2020 when a substantial decline (19.9%) was observed. This sharp decrease in TB incidence might have been related to multiple factors coinciding with the COVID-19 pandemic, including delayed or missed TB diagnoses or a true reduction in TB incidence related to pandemic mitigation efforts and changes in immigration and travel (*2*). During 2021, a total of 7,860 TB cases were provisionally reported to CDC’s National Tuberculosis Surveillance System (NTSS) by the 50 U.S. states and the District of Columbia (DC). National incidence of reported TB (cases per 100,000 persons) rose 9.4% during 2021 (2.37) compared with that in 2020 (2.16) but remained 12.6% lower than the rate during 2019 (2.71).* During 2021, TB incidence increased among both U.S.-born and non–U.S.-born persons. The increased TB incidence observed during 2021 compared with 2020 might be partially explained by delayed diagnosis of cases in persons with symptom onset during 2020; however, the continued, substantial reduction from prepandemic levels raises concern for ongoing underdiagnosis. TB control and prevention services, including early diagnosis and complete treatment of TB and latent TB infection, should be maintained and TB awareness promoted to achieve elimination in the United States.

Health departments in the 50 U.S. states and DC report TB cases to CDC based on the Council of State and Territorial Epidemiologists’ surveillance case definition, which includes both laboratory and clinically verified cases.^†^ For each case, health departments electronically submit a report of a verified TB case to CDC. Midyear U.S. Census Bureau population estimates^§^ were used to calculate national- and state-level TB incidence per 100,000 persons along with incidence stratified by age groups. Persons with TB were grouped by self-reported race and ethnicity according to federal guidelines.^¶^ Persons who self-identified as Hispanic were categorized as Hispanic irrespective of self-reported race, persons not identifying as Hispanic were categorized by self-reported race, and non-Hispanic persons who reported more than one race were categorized as “multiple races.” Midyear population estimates from the Current Population Survey** were used to calculate incidence by birth origin^††^ and race/ethnicity. Percent changes in incidence were calculated using unrounded figures.

A total of 7,860 TB cases were reported during 2021, 687 more than during 2020 (7,173) and 1,040 fewer than during 2019 (8,900) (Table 1). From 2020 to 2021, TB incidence (cases per 100,000 population) rose 9.4%, from 2.16 to 2.37, but remained 12.6% lower than during 2019 (2.71). California reported the highest number of cases (1,750), and Alaska reported the highest incidence (7.92). Eighteen states and DC reported the same number or fewer TB cases during 2021 than during 2020; the remaining 32 states reported more cases during 2021 than 2020.

**TABLE 1:** Tuberculosis disease case counts and incidence, by U.S. state — 50 states and the District of Columbia, 2019–2021

U.S. jurisdiction	No. of TB cases*			TB incidence^†^		
	2019	2020	2021	2019	2020	2021
**Total**	**8,900**	**7,173**	**7,860**	**2.71**	**2.16**	**2.37**
Alabama	87	72	92	1.77	1.43	1.83
Alaska	58	58	58	7.91	7.92	7.92
Arizona	183	136	129	2.51	1.89	1.77
Arkansas	64	59	69	2.12	1.96	2.28
California	2,111	1,706	1,750	5.35	4.32	4.46
Colorado	66	52	58	1.15	0.90	1.00
Connecticut	67	54	54	1.88	1.50	1.50
Delaware	18	17	43	1.84	1.71	4.29
District of Columbia	24	19	19	3.39	2.75	2.84
Florida	558	412	499	2.60	1.91	2.29
Georgia	302	221	228	2.84	2.06	2.11
Hawaii	99	92	106	6.99	6.34	7.35
Idaho	7	8	4	0.39	0.43	0.21
Illinois	326	216	255	2.57	1.69	2.01
Indiana	108	92	127	1.60	1.36	1.87
Iowa	52	39	49	1.65	1.22	1.53
Kansas	37	37	43	1.27	1.26	1.47
Kentucky	66	67	57	1.48	1.49	1.26
Louisiana	88	99	86	1.89	2.13	1.86
Maine	18	17	14	1.34	1.25	1.02
Maryland	209	148	192	3.45	2.40	3.11
Massachusetts	178	142	151	2.58	2.02	2.16
Michigan	131	101	136	1.31	1.00	1.35
Minnesota	148	117	134	2.62	2.05	2.35
Mississippi	58	41	45	1.95	1.39	1.53
Missouri	70	79	77	1.14	1.28	1.25
Montana	2	4	3	0.19	0.37	0.27
Nebraska	17	33	22	0.88	1.68	1.12
Nevada	53	57	61	1.71	1.83	1.94
New Hampshire	6	12	12	0.44	0.87	0.86
New Jersey	310	245	279	3.49	2.64	3.01
New Mexico	41	29	24	1.95	1.37	1.13
New York	746	605	681	3.83	3.00	3.43
North Carolina	185	159	178	1.76	1.52	1.69
North Dakota	18	10	15	2.36	1.28	1.94
Ohio	150	130	149	1.28	1.10	1.26
Oklahoma	73	67	69	1.84	1.69	1.73
Oregon	70	67	78	1.66	1.58	1.84
Pennsylvania	199	156	166	1.55	1.20	1.28
Rhode Island	14	7	17	1.32	0.64	1.55
South Carolina	80	67	88	1.55	1.31	1.70
South Dakota	16	16	12	1.80	1.80	1.34
Tennessee	129	113	85	1.89	1.63	1.22
Texas	1,154	883	991	3.98	3.02	3.36
Utah	27	29	17	0.84	0.88	0.51
Vermont	4	3	2	0.64	0.47	0.31
Virginia	191	169	161	2.23	1.96	1.86
Washington	221	163	199	2.90	2.11	2.57
West Virginia	9	13	7	0.50	0.73	0.39
Wisconsin	51	35	66	0.88	0.59	1.12
Wyoming	1	0	3	0.17	0.00	0.52

**Abbreviation**: TB = tuberculosis.

* Case counts are based on data reported to the National Tuberculosis Surveillance System as of February 9, 2022.

^†^ Cases per 100,000 persons using midyear population estimates from the U.S. Census Bureau. 2019 population estimates are based on the 2010 U.S. Census. 2020 and 2021 population estimates are based on the 2020 U.S. Census. https://www.census.gov/programs-surveys/popest/data/tables.html

During 2021, 71% of TB cases occurred among non–U.S.-born persons, the same proportion as in 2020 and 2019. Incidence (cases per 100,000 population) among U.S.-born persons increased from 0.71 in 2020 to 0.79 in 2021 and among non-U.S.-born persons from 11.71 in 2020 to 12.16 in 2021 (Figure). Among U.S.-born persons reported as having TB disease, 4% identified as American Indian or Alaska Native (AI/AN), 6% as Asian, 33% as Black, 25% as Hispanic, 2% as Native Hawaiian or other Pacific Islander (NH/OPI), 29% as White, and 1% as multiple races.^§§^ From 2020 to 2021, TB incidence decreased 0.4% among U.S.-born Black persons and 5.7% among U.S.-born NH/OPI persons and increased among all other U.S.-born groups (including AI/AN [5.0%], Asian [32.6%], Hispanic [16.3%], and White [13.8%] persons) (Table 2). Among non–U.S.-born persons reported as having TB disease, <1% identified as AI/AN, 48% as Asian, 12% as Black, 34% as Hispanic, 1% as NH/OPI, 4% as White, and 1% as multiple races. From 2020 to 2021, TB incidence decreased 8.7% among non–U.S.-born Black persons and 40.3% among non–U.S.-born NH/OPI persons and increased among all other non–U.S.-born groups (including Asian [3.7%], Hispanic [7.9%], and White [4.5%] persons).^¶¶^ Compared with TB incidence in 2020, incidence during 2021 declined 2.2% among children aged ≤4 years, 0.3% among children and adolescents aged 5–14 years, and 2.9% among persons aged 15–24 years. Incidence increased among adults aged 25–44 years (5.3%), 45–64 years (10.6%), and ≥65 years (13.2%).

**FIGURE:**
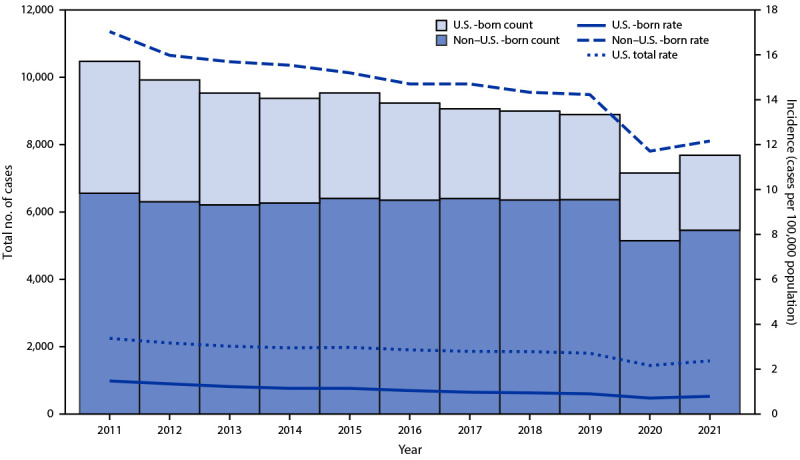
Tuberculosis disease case counts* and incidence,**^†^** by patient birth origin**^§^** — United States, 2011–2021 * Case counts are based on data from the National Tuberculosis Surveillance System as of February 9, 2022. ^†^ Cases per 100,000 persons. The Current Population Survey provides the population denominators used to calculate tuberculosis incidence according to national origin and racial/ethnic group. https://www.census.gov/programs-surveys/cps.html (Accessed February 9, 2022). ^§^ Cases with unknown origin at birth excluded.

**TABLE 2:** Tuberculosis disease case counts and incidence, by birth origin and race/ethnicity — United States, 2018–2021

Birth origin and race/ethnicity	No. of TB cases* (incidence^†^)			
	2018	2019	2020	2021
**U.S.-born^§^**				
AI/AN	100 (3.91)	79 (3.35)	78 (3.54)	84 (3.72)
Asian	135 (1.88)	115 (1.50)	95 (1.15)	125 (1.53)
Black, non-Hispanic	950 (2.67)	909 (2.56)	731 (2.04)	739 (2.03)
Hispanic	584 (1.47)	609 (1.52)	475 (1.17)	553 (1.36)
White, non-Hispanic	806 (0.43)	756 (0.41)	565 (0.30)	640 (0.35)
NH/OPI	39 (5.17)	26 (3.92)	42 (6.20)	45 (5.85)
White, non-Hispanic	806 (0.43)	756 (0.41)	565 (0.30)	640 (0.35)
Unknown race/ethnicity or multiple races^¶^	28 (—)	31 (—)	21 (—)	38 (—)
**Subtotal**	**2,642 (0.95)**	**2,525 (0.90)**	**2,007 (0.71)**	**2,224 (0.79)**
**Non–U.S.-born**				
AI/AN	2 (3.49)	2 (3.51)	0 (—)	1 (1.33)
Asian	3,074 (26.08)	3,049 (26.17)	2,478 (22.18)	2,613 (22.99)
Black, non-Hispanic	848 (20.36)	838 (19.83)	679 (15.67)	623 (14.30)
Hispanic	2,040 (10.28)	2,076 (10.24)	1,650 (8.17)	1,821 (8.81)
NH/OPI	73 (24.70)	80 (24.78)	75 (35.36)	68 (21.10)
White, non-Hispanic	260 (3.23)	254 (3.16)	212 (2.73)	225 (2.85)
Unknown race/ethnicity or multiple races^¶^	58 (—)	70 (—)	55 (—)	105 (—)
**Subtotal**	**6,355 (14.33)**	**6,368 (14.23)**	**5,149 (11.71)**	**5,456 (12.16)**
Unknown national origin^¶^	3 (—)	7 (—)	17 (—)	180 (—)
**Total**	**9,000 (2.75)**	**8,900 (2.71)**	**7,173 (2.16)**	**7,860 (2.37)**

**Abbreviations**: AI/AN = American Indian or Alaska Native; NH/OPI = Native Hawaiian or other Pacific Islander; TB = tuberculosis.

*Case counts are based on data from the National Tuberculosis Surveillance System as of February 9, 2022.

^†^ Incidence is calculated per 100,000 persons. The Current Population Survey (https://www.census.gov/programs-surveys/cps.html) provides the population denominators used to calculate TB incidence according to national origin and racial/ethnic group. (Accessed February 9, 2022). Total rate was calculated by using midyear population estimates from the U.S. Census Bureau.

^§^ Persons born in the United States or a U.S. territory or elsewhere to at least one U.S. citizen parent are categorized as U.S.-born. All other persons are categorized as non–U.S.-born.

^¶^ Incidence rates were not calculated for these categories because population estimates were not available.

During 2021, among non–U.S.-born persons reported as having TB, 9.3% (507 of 5,456) received a diagnosis <1 year after arrival in the United States, compared with 9.7% (499 of 5,149) during 2020 and an average of 15.6% (996 of 6,377) during 2015–2019. Among non–U.S.-born persons with reported TB during 2021, approximately one third (1,811; 33.2%) had lived in the United States for at least 20 years before receiving a diagnosis, similar to the percentage during 2020 (1,662; 32%), and slightly more than the average of 28% (1,766) during 2015–2019. The proportion of persons who received a diagnosis of TB who had visible acid-fast bacilli on sputum smear microscopy, a marker of infectiousness and more advanced disease, during 2020 (46.4%) and 2021 (48.1%) were higher than the average proportion during 2015–2019 (44.3%).*** When stratified by birth origin, the prevalence of smear positivity among non–U.S.-born persons during 2020 (45.5%) and 2021 (47.8%) were higher than the average during 2015–2019 (42.6%). This increase in smear-positivity was not observed among U.S.-born persons who had received a diagnosis of TB (2021 = 48.2%; 2020 = 48.9%; average 2015–2019 = 48.7%).

## Discussion

U.S. TB incidence during 2021 increased by 9.4% following a large decrease during 2020 (*2*). Although TB cases and incidences have gradually declined in the United States since 1993, with a slowing pace of decline in recent years (*1*), larger changes in reported TB have occurred during the COVID-19 pandemic. Similar changes in TB incidence have been reported globally (*3*,*4*). In the United States, the causes for the changes in TB incidence are likely multifactorial. Probable explanations include a true reduction in TB disease resulting from reduced TB transmission because of pandemic mitigation efforts and fewer new arrivals from countries with higher TB incidence than the United States. In addition, delayed or missed TB diagnoses because of disruptions in health care access or assumptions that patients with respiratory symptoms had COVID-19 might contribute to the observed changes (*5*).

The reduction in the number of persons with TB disease reported <1 year after arrival in the United States coincides with changes in immigration and travel associated with the pandemic. Immigration to the United States declined by 31% during 2020,^†††^ and similar patterns are suggested during 2021.^§§§^ However, immigration and travel reductions during 2020–2021 cannot fully account for the reduction in TB, because most TB cases among non–U.S.-born persons occur among those who have lived in the United States for many years and are likely the result of reactivation of latent TB infection (LTBI) (*1*). Despite overall case count declines, the number of TB cases among non–U.S.-born persons living in the United States for 20 years or longer before diagnosis increased during 2021 compared with average case counts during 2015–2019, highlighting the importance of evaluation and treatment of LTBI to prevent progression to TB disease. CDC is working to raise awareness of TB and LTBI among communities at risk and their health care providers through the new “Think. Test. Treat TB” campaign.^¶¶¶^

The increased TB incidence observed during 2021 compared with 2020 might be partially explained by delayed detection of cases with symptom onset during 2020 that were not diagnosed until 2021 because of delayed health care–seeking behavior, interruptions in health care access, or disrupted TB services related to the COVID-19 pandemic (*6*,*7*). The small increase in the prevalence of smear positivity at diagnosis, predominantly among non–U.S.-born persons, suggests more advanced pulmonary disease, which might result from delayed diagnosis. Avoiding missed or delayed diagnosis of TB is crucial to preventing transmission. TB should be considered in the differential diagnosis of patients with prolonged cough (>2 weeks) or TB symptoms such as unintentional weight loss or hemoptysis, particularly among persons with epidemiologic risk factors for TB (e.g., birth or former residence in a country with higher TB incidence than that in the United States, history of living in a congregate setting such as a homeless shelter or a correctional facility, or immune suppression).****

The findings in this report are subject to at least two limitations. First, this analysis is limited to provisional 2021 TB surveillance data and case counts might change. Second, calculated rates are based on population estimates that are subject to change.

Ongoing analyses of NTSS data and external data sources, including anti-TB drug dispensing and hospitalization data, will provide more information about the effects of the COVID-19 pandemic on U.S. TB epidemiology, including the extent to which delayed diagnosis has been a factor. Focusing on essential TB activities, including early diagnosis and complete treatment of TB and LTBI, remains critical to achieving TB elimination in the United States.

**Summary**

**What is already known about this topic?**

The number of reported U.S. tuberculosis (TB) cases decreased sharply in 2020, possibly related to multiple factors associated with the COVID-19 pandemic.

**What is added by this report?**

Reported TB incidence (cases per 100,000 persons) increased 9.4%, from 2.2 during 2020 to 2.4 during 2021 but was lower than incidence during 2019 (2.7). Increases occurred among both U.S.-born and non–U.S.-born persons.

**What are the implications for public health practice?**

Factors contributing to changes in reported TB during 2020–2021 likely include an actual reduction in TB incidence as well as delayed or missed TB diagnoses. Timely evaluation and treatment of TB and latent tuberculosis infection remain critical to achieving U.S. TB elimination.

## Footnotes

* This report is limited to National Tuberculosis Surveillance System data verified as of February 9, 2022. Updated data will be available in CDC’s annual TB surveillance report later in 2022.

†
https://www.cdc.gov/tb/programs/rvct/instructionmanual.pdf

§ 2021 vintage population estimates were used for 2021 and 2020. 2020 vintage population estimates were used for 2011–2019. https://www.census.gov/programs-surveys/popest/data/tables.html

¶
https://www.census.gov/topics/population/race/about.html

**
https://www.census.gov/programs-surveys/cps.html

†† Persons born in the United States or a U.S. territory or elsewhere to at least one U.S. citizen parent are categorized as U.S.-born. All other persons are categorized as non–U.S.-born.

§§ Persons with missing race or ethnicity data are excluded from calculations of proportions.

¶¶ Percent change is not reported for non–U.S.-born AI/AN persons because there were no reported cases during 2020.

*** Percentage of positive sputum smears is calculated among persons with a positive or negative sputum smear result; those with unknown results or for whom testing was reported as not performed were excluded.

†††
https://www.dhs.gov/immigration-statistics/yearbook/2020

§§§
https://www.census.gov/library/stories/2021/12/net-international-migration-at-lowest-levels-in-decades.html

¶¶¶
https://www.cdc.gov/thinktesttreattb/

**** Clinical consultation for potential TB cases is also available through state or local TB programs or the CDC-sponsored TB Centers of Excellence. https://www.cdc.gov/tb/education/professionaltools.htm
